# Molecular-Level Investigation of Cycloaliphatic Epoxidised Ionic Liquids as a New Generation of Monomers for Versatile Poly(Ionic Liquids)

**DOI:** 10.3390/polym13091512

**Published:** 2021-05-07

**Authors:** Baris Demir, Gabriel Perli, Kit-Ying Chan, Jannick Duchet-Rumeau, Sébastien Livi

**Affiliations:** 1 Centre for Theoretical and Computational Molecular Science, The Australian Institute for Bioengineering and Nanotechnology, The University of Queensland, Brisbane, QLD 4072, Australia; 2 Ingénierie des Matériaux Polymères, Université de Lyon, CNRS, UMR 5223, INSA Lyon, F-69621 Villeurbanne, France; gabriel.perli@insa-lyon.fr (G.P.); jannick.duchet@insa-lyon.fr (J.D.-R.); sebastien.livi@insa-lyon.fr (S.L.); 3 Department of Mechanical and Aerospace Engineering, The Hong Kong University of Science and Technology, Hong Kong, China; chankitying@ust.hk

**Keywords:** poly(ionic liquids), ionic liquids, polymerisation, thermo–mechanical properties, molecular dynamics simulations

## Abstract

Recently, a new generation of polymerised ionic liquids with high thermal stability and good mechanical performances has been designed through novel and versatile cycloaliphatic epoxy-functionalised ionic liquids (CEILs). From these first promising results and unexplored chemical structures in terms of final properties of the PILs, a computational approach based on molecular dynamics simulations has been developed to generate polymer models and predict the thermo–mechanical properties (e.g., glass transition temperature and Young’s modulus) of experimentally investigated CEILs for producing multi-functional polymer materials. Here, a completely reproducible and reliable computational protocol is provided to design, test and tune poly(ionic liquids) based on epoxidised ionic liquid monomers for future multi-functional thermoset polymers.

## 1. Introduction

Prepolymers based on cycloaliphatic epoxy monomers present several advantages compared to diglycidyl ether derivative monomers, namely low viscosity, high electrical resistivity, and UV resistance [1,2,3,4,5,6]. Considering these outstanding features, numerous works have investigated cycloaliphatic epoxy precursors for different applications including the coating of electronic components, electrical insulation, and the development of optical devices [7,8,9,10,11]. Owing to their higher chemical reactivity compared to Bisphenol A diglycidyl ether (DGEBA), cycloaliphatic epoxy monomers can go through a homopolymerisation pathway under thermal or photoinitiated conditions. Remarkably, the possibility of triggering their polymerisation by light allows the employment of these monomers for the development of 3D printing technologies, like stereolithography. However, in addition to all these outstanding possibilities, epoxy-based materials are extremely brittle due to the more compact structure of the network formed [12,13,14,15]. For these reasons, a lot of effort has been made to tune the mechanical properties of these materials. In this context, the design and synthesis of epoxy monomers bearing ionic liquid (IL) moieties emerge as an innovative alternative. Indeed, incorporating IL segments into the monomer backbone must result in a polymer with particular properties [16,17,18,19,20]. Radchenko et al. synthesised new cycloaliphatic epoxidised ILs to develop a novel generation of poly(ionic liquids) (PILs) [20]. In their work, they have detailed the polymerisation mechanism of new ILs containing epoxidised cycloaliphatic groups, denoted as cycloaliphatic epoxy-functionalised ionic liquids (CEILs). Although three epoxidised ILs have been synthesised, the study focused mainly on the development and characterisation of a PIL based on one of the monomers, leaving further research avenues for future for works.

Notwithstanding the fact that the design of epoxidised ILs is an elegant and efficient strategy to tailor new epoxy materials, the synthesis of monomers can be costly and laborious. Hence, the development of computational routines that are able to accurately predict the mechanical properties of materials can supply valuable insights for monomer designing [21]. Molecular dynamics (MD) simulations are a versatile tool to investigate materials at the molecular level. Several groups developed computational protocols to make, test and tune thermoset polymers for epoxies [22,23,24,25,26,27,28,29]. For example, Komarov et al. performed multiscale simulations to generate cycloaliphatic epoxy resin networks based on ECC and 4-methylhexahydrophthalic anhydride (MHHPA-4) [22]. Thermal properties such as glass transition temperature and the coefficient of thermal expansion were predicted as a function of the conversion of monomers. Komarov et al. utilised Monte Carlo simulations to obtain relaxed and equilibrated polymer samples. Chinkanjanarot et al. investigated a cycloaliphatic epoxy resin system where epoxy-cyclohexyl-methyl-3,4-epoxy-cyclohexyl-carboxylate (EEC) was cured with an anhydride curing agent [25]. These authors reported predicted thermo–mechanical properties and the thermal conductivity of their model polymers. In a similar work, Radue et al. developed a computational algorithm to capture the complex curing mechanisms of Matrimid-5292 and predict such macroscopic properties such as the coefficient of thermal expansion and Young’s modulus as a function of monomer conversion using MD simulations [26].

Following the work of Livi, Radchenko et al. [20], we decided to reveal the structure–property relationship in CEILs using MD simulations. The polymerisation procedure reported in the literature [24] has been adapted to model the cationic polymerisation of CEILs and their characterisation at the molecular level. The procedure relies on capturing the bond formation between monomers on the fly. In other words, bonds between monomers form during the simulations. Our computational procedure is based on the following steps. First, a mixture of cation (epoxy monomer)–anion for each CEIL was prepared and adequately equilibrated. Second, the equilibrated samples were polymerised using a polymerisation protocol that was adapted from a procedure, which was previously developed to model epoxy polymers [24]. Third, the polymerised samples were used to predict thermo–mechanical properties (e.g., glass transition temperature and Young’s modulus). Finally, the mobility of anions in the polymerised samples were predicted to assess the suitability of CEILs to be used as solid electrolytes in energy applications. In addition, the thermal stabilities of ILs were assessed by thermogravimetric analysis (TGA). The thermo–mechanical properties of the polymerised samples were experimentally measured via differential scanning calorimetry (DSC) and dynamic mechanical analysis (DMA). The computational protocol reported herein can be translated to model CEILs based on epoxy-functionalised ILs with varying architecture and functionality. Our protocol is entirely reproducible and can provide a platform for making, testing and tuning PILs with desired properties.

## 2. Materials and Methods

### 2.1. Computational Methods

#### 2.1.1. Preparation of Initial Liquid Precursors

All-atom molecular dynamics (MD) simulations were carried out using the Dreiding force field [30]. We applied periodic boundary conditions in all three dimensions. The Nosé–Hoover thermostat [31,32] with a time constant of 100 fs and a barostat [31,33] with a time constant of 1000 fs were implemented in the NVT-MD (only thermostat) and NPT-MD (both thermostat and barostat) simulations. A cut-off distance of 12 Å was used for the long-range van der Waals (vdW) interactions. Coulomb interactions were treated with long-range particle–particle–particle–mesh (PPPM) solver [34] with a real space cut-off of 12 Å and a precision of 10${}^{-4}$. Newton’s equations of motion were integrated with a time-step of 1 fs, using the open source molecular simulation tool, LAMMPS (lammps.sandia.gov) [35]. Visual molecular dynamics (VMD) was used to visualise the trajectories of samples [36].

**Figure 1:**
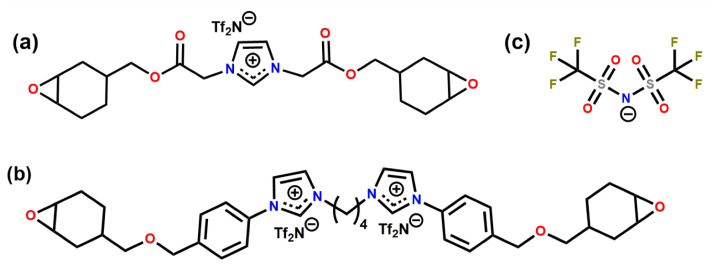
Chemical structures of (**a**) CEIL-2 monomer, (**b**) CEIL-3 monomer and (**c**) Tf${}_{2}$N${}^{-}$.

We generated the initial structures of the ions using the AVOGADRO software package [37]. Once the initial structures were geometry optimised, we calculated the partial atomic charges of the atoms following the procedure reported previously [24]. The partial charges are reported in SI. We tested two experimentally investigated systems: CEIL-2 and CEIL-3 [20]. Each system consists of one type of cation and one type of anion. In CEIL-2, the ratio between the positively charged ions (i.e., the monomers) and negatively charged ions (anions) is one as there is only one imidazolium ring present in the monomer. In CEIL-3, this ratio is 0.5. In other words, we have one cation consisting of two imidazolium rings and two anions. We used the same type of anion for both systems. Figure 1 shows the molecular sketches for monomers and the anion.

We generated liquid samples by randomly packing the activated monomer and anion [38] using the PACKMOL software package [39]. What is meant by “activated monomer” is that the epoxide bond located on both ends of each monomer is broken, and the resulting unsaturated carbon and oxygen atoms are saturated with hydrogen. The monomer and ions were randomly packed in a cubic simulation cell with a dimension of 200 Å. The initial density of liquid mixtures was deliberately set to a low density value in order to avoid any potential atomic overlaps during the random packing of monomers and anions in the simulation cells. Table 1 shows the numbers of monomers and anions as well as the total number of atoms for each liquid mixture sample. We then geometrically optimised the liquid precursor mixtures using the FIRE algorithm [40] for 50,000 steps. Following this, the liquid samples were equilibrated at 227 ${}^{{}^{\circ}}$C. An NVT-MD simulation was performed for a period of 150 ps. We then equilibrated the density of the simulation cells by performing an isotropic NPT-MD simulation at 227 ${}^{{}^{\circ}}$C and 1 atm for 300 ps. We chose an elevated temperature of 227 ${}^{{}^{\circ}}$C to ensure that the monomers and anions had sufficient mobility for adequate mixing.

Once we obtained the equilibrium density of liquid mixtures at 227 ${}^{{}^{\circ}}$C and 1 atm, we applied a simulated annealing (SA) procedure [24]. In this procedure, the system temperature was varied between 227 and 727 ${}^{{}^{\circ}}$C via the use of NVT-MD simulations. We first simulated the samples at 227 ${}^{{}^{\circ}}$C for a period of 0.2 ns and then heated them to 727 ${}^{{}^{\circ}}$C for 0.2 ns. We then kept the samples at 727 ${}^{{}^{\circ}}$C for 1.0 ns. Following this, the liquid samples were cooled down to 227 ${}^{{}^{\circ}}$C over a period of 0.5 ns. We then performed a simulation for 0.2 ns and recorded the resultant trajectory at the end of each 1 ps for analysis. We used the trajectories to calculate the radial distribution functions (RDFs), which provide the information about the formation of molecular-level structuring in the liquid samples. The oxygen atom of the broken epoxide bonds located on both ends of each monomer and the nitrogen atom of the anion (i.e., Tf${}_{2}$N${}^{-}$) were used as reference points in the RDF calculations. We report the properties averaged over three independently generated samples.

#### 2.1.2. Polymerisation Procedure

**Figure 2:**
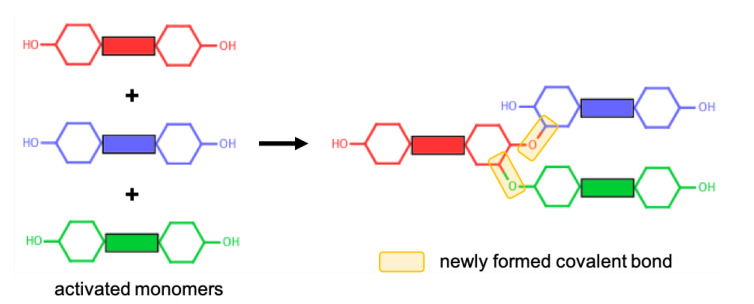
A representative reaction mechanism taking place between monomers.

Polymerisation of cycloaliphatic epoxy systems occurs via the cationic polymerisation of monomers. Since we used an activated form of monomers, no ring opening reaction was modelled during the polymerisation procedure. A reactive oxygen atom of any monomer (i.e., the oxygen atom of the hydroxyl group) can react with a reactive carbon atom of any monomer (i.e., the carbon atom that was initially connected to an oxygen atom that is now a reactive oxygen atom). The reaction mechanism for the cationic polymerisation of monomers between the reactive atoms of representative CEILs is shown in Figure 2. Once a target degree of polymerisation (DOP) was achieved, the excess hydrogen atoms connected to the reacted oxygen and carbon atoms were deleted. The liquid samples were polymerised until a target DOP of >99% was achieved. We define DOP as the ratio between the reacted reactive oxygen atoms of the monomers and the total number of reactive oxygen atoms of the monomers present in the simulation cell prior to the polymerisation process. In addition, we updated the partial atomic charges of atoms formed a covalent bond during the polymerisation process. The updated charges were calculated using the procedure reported in the literature [24]. Briefly, only reacted oxygen and carbon atoms were assigned new charges as their chemical environment altered after polymerisation. We reported the updated partial charges in SI. We should note here that the anions in both systems do not chemically contribute to the polymer network formed during the polymerisation process. Anions are free to move in the polymer matrix.

#### 2.1.3. Prediction of Thermo–Mechanical Properties

Once the polymerisation procedure was completed, thermo–mechanical properties of the polymer samples (p-CEIL-2 and p-CEIL-3) such as glass transition temperature (T${}_{g}$), coefficients of volumetric thermal expansions ($\alpha_{v}$) and coefficients of linear thermal expansions ($\alpha_{l}$) were calculated. We name the liquid samples CEIL-2 and CEIL-3, while the polymerised samples are named p-CEIL-2 and p-CEIL-3. We calculated the T${}_{g}$ of the polymerised samples via the use of isotropic isobaric cooling (i.e., the isotropic NPT-MD). The samples were cooled from 327 to 27 ${}^{{}^{\circ}}$C with a cooling rate of 20 ${}^{{}^{\circ}}$C·ns${}^{-1}$. Each sample was cooled at intervals of 10 ${}^{{}^{\circ}}$C, and simulated for 0.5 ns at each temperature point, amounting to a total of 15.5 ns of MD simulation. We then calculated the density at each temperature point (an interval of 10 ${}^{{}^{\circ}}$C) for each sample and averaged over three independently generated samples for each polymer system. A piece-wise data fitting method was applied to determine the value of T${}_{g}$. In this method, two lines were fit to the temperature versus density data, and the intersection of these two lines was determined as the T${}_{g}$. It should be noted here that the cooling rate implemented in our simulations is many orders of magnitude larger than the experimentally achievable cooling rates. A typical exercise to overcome this discrepancy is to subtract 3 ${}^{{}^{\circ}}$C from the predicted T${}_{g}$s per order of magnitude in difference between the cooling rates [41]. Later, the $\alpha_{v}$ was predicted for both glassy and rubbery states for each polymerised system. The slope of each line fit to the volume fraction versus temperature plot was determined as $\alpha_{v}$ value. The volume fraction at temperature *T* was calculated as V(T)−V(27))/V(27). We can also predict that the $\alpha_{l}$ for isotropic structures (i.e., the property of a material is the same in all the directions) by simply taking one-third of the $\alpha_{v}$ value.

Tensile stress–strain curves (SSCs) can be used to predict the Young’s modulus of a material. We deformed our samples under tensile stress by applying a constant strain of 5 × 10${}^{7}$ s${}^{-1}$ in one direction while keeping the cell dimensions in the other two directions free to change. The deformation tests were performed at 27 ${}^{{}^{\circ}}$C and 1 atm. We averaged the tensile stress and strain data over each 1000 steps to obtain the tensile SSC for each run. We performed tensile deformation simulations in each direction separately. We then averaged the stress and strain data over each direction and three samples per system. In total, nine simulations were performed per system. Young’s modulus was calculated from the slope of the averaged tensile SSCs. The 2.5% strain was used to determine the slope of the tensile SSC (or the Young’s modulus).

In addition, Poisson’s ratio, (ν) was calculated for each system. Poisson’s ratio is defined as the ratio between the normalised decrease in the lateral dimensions of the simulation cell ($\varepsilon_{lateral}$) and the normalised increase in the dimension of the simulation cell where the tensile strain is applied ($\varepsilon_{axial}$). We then calculated Poisson’s ratio from the slope of the line fit to the strain in the direction of tensile deformation versus the strain averaged over the other two directions:

(1) $$\nu =-\frac{d\varepsilon_{lateral}}{d\varepsilon_{axial}}$$

#### 2.1.4. Mobility of Anions

Upon the completion of thermo–mechanical analysis, we examined the mobility of anions in the polymerised samples. As mentioned previously, anions do not chemically contribute to the polymer network and can freely move in the polymerised samples. To examine the mobility of anions, we performed an NVT-MD simulation for 10 ns at 27 ${}^{{}^{\circ}}$C for each sample of each system. For each sample, we performed three sets of simulations for 10 ns. A different set of velocities were assigned to the atoms of the sample, and a simulation of 0.2 ns in the NVT-MD was run before the 10 ns production run. Therefore, a total duration of 90 ns production simulation was performed for each system. Three-dimensional mean squared displacement (MSD) curves for the anion (i.e., Tf${}_{2}$N${}^{-}$) were calculated using the following equation:

(2) $$MSD=\frac{1}{N}\sum_{i=1}^{N}\langle {\left(\vec{r_{i}}\left(t\right)-\vec{r_{i}}\left(0\right)\right)}^{2}\rangle$$

where $\vec{r_{i}}\left(t\right)$ and $\vec{r_{i}}\left(0\right)$ represent the position of atom *i* at time *t* and time 0, respectively. We considered the nitrogen atom of Tf${}_{2}$N${}^{-}$ as the representative site in the MSD calculations.

### 2.2. Experimental Method

#### 2.2.1. Synthesis of IL Monomers

All reagents were purchased from Sigma-Aldrich or TCI and were used without any further purification. All the solvents were acquired as anhydrous from Sigma Aldrich. Both ILs were synthesised in high yields and at a large scale, based on a methodology described in our previous article [20]. The monomer CEIL-2 was obtained as a white solid, while CEIL-3 corresponds to a colourless and transparent viscous oil. Both ILs and their intermediates were characterised by ${}^{1}$H, ${}^{19}$F, and ${}^{13}$C NMR analysis.

#### 2.2.2. Epoxy Network Preparation

To prepare epoxy networks, CEIL-2 was dissolved in dichloromethane at room temperature. The obtained mixture was poured into silicone moulds, and the solvent was then slowly removed under vacuum. The CEIL-3 derived network was obtained adding the monomer directly to the silicone mould and leaving it to degas under vacuum. Subsequently, the moulds were placed in the oven at 100 ${}^{{}^{\circ}}$C for 1 h to liquidise the resin and fit the mould properly. Then, the temperature was increased up to 150 ${}^{{}^{\circ}}$C for 5 h with a final step at 240 ${}^{{}^{\circ}}$C for 1 h. The efficacy of the curing protocol was confirmed by differential scanning calorimetry (DSC) and dynamic mechanical analysis (DMA).

#### 2.2.3. Experimental Analyses

The thermal stabilities of both neat ionic liquid epoxy monomers were evaluated by the Q500 thermogravimetric analyzer (TGA) (TA Instruments). Five samples for each system were tested. The samples were heated from 25 to 700 ${}^{{}^{\circ}}$C at a rate of 10 ${}^{{}^{\circ}}$C·min${}^{-1}$ under nitrogen flow with a purge flow rate of 90 mL·min${}^{-1}$. The differential scanning calorimetry (DSC) analyses of the epoxy networks were performed on a Q10 (TA instruments) in a dynamic mode from 0 to 200 ${}^{{}^{\circ}}$C at a rate of 10 ${}^{{}^{\circ}}$C·min${}^{-1}$ under nitrogen flow of 50 mL·min${}^{-1}$. The mechanical material properties were investigated by torsional mode using an ARES−G2 rheometer (TA Instruments). Rectangular samples were prepared with dimensions of 30 × 6 × 3 mm${}^{3}$ and the measurements were carried out under a frequency of 1 Hz. Storage modulus G${}^{\prime }$, loss modulus G${}^{\prime \prime }$ and loss factor tanδ were assessed in the interval of temperature from −100 to 180 ${}^{{}^{\circ}}$C with a heating rate of 3 ${}^{{}^{\circ}}$C·min${}^{-1}$.

## 3. Results and Discussion

We obtained the computational structural and macroscopic properties using the results averaged over three independent samples for each system. The liquid samples were named CEIL-2 and CEIL-3, while a prefix “p-” was used to name the polymerised samples, p-CEIL-2 and p-CEIL-3.

### 3.1. Modelling of Liquid Samples

**Figure 3:**
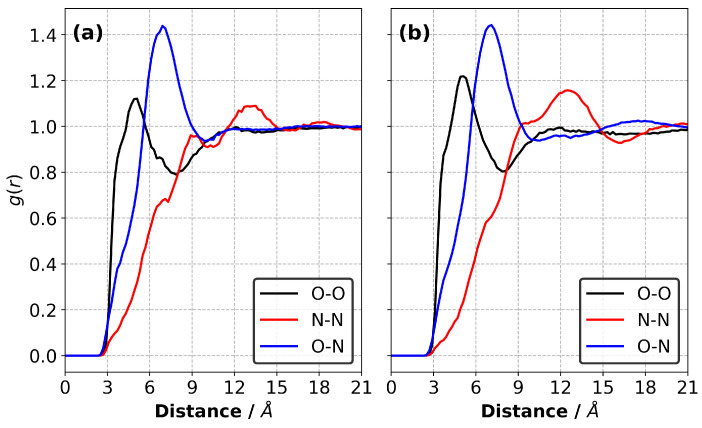
Radial distribution functions (RDFs) between the monomers (the reactive oxygen atom was chosen as a representative atomic site) and anions (the nitrogen atom of the Tf${}_{2}$N${}^{-}$ was chosen as a representative atomic site) in the liquid (**a**) CEIL-2 and (**b**) CEIL-3 systems at 227 ${}^{{}^{\circ}}$C.

We calculated the radial distribution functions (RDFs) for the atomic pairs between the monomers, anions and monomers–anions in the equilibrated liquid precursor mixtures at 227 ${}^{{}^{\circ}}$C, as shown in Figure 3. Our results show no substantial differences between the RDFs obtained for the CEIL-2 and CEIL-3 liquid mixtures. We found that the most likely distance between the reactive atomic sites on the monomer (i.e., the first peak of the black curves in Figure 3) was ∼4.9 Å for both systems. This is a smaller distance than the most likely distance between the reactive oxygen atom of the monomers and nitrogen atom of the anions (i.e., the first peak of the blue curves in Figure 3), which was ∼6.9 Å. The RDFs calculated between the anions (i.e., the first peak of the red curves in Figure 3) show two shoulders located ∼6.9 Å and ∼9.0 Å. A peak corresponding to an amplitude larger than unity was found to be ∼13.5 Å and ∼12.3 Å for the CEIL-2 and CEIL-3 systems, respectively. These results show that anions do not prefer to be found close to each other, suggesting unfavoured Coulombic interactions between the anions.

### 3.2. Thermal Stabilities of CEILs

**Figure 4:**
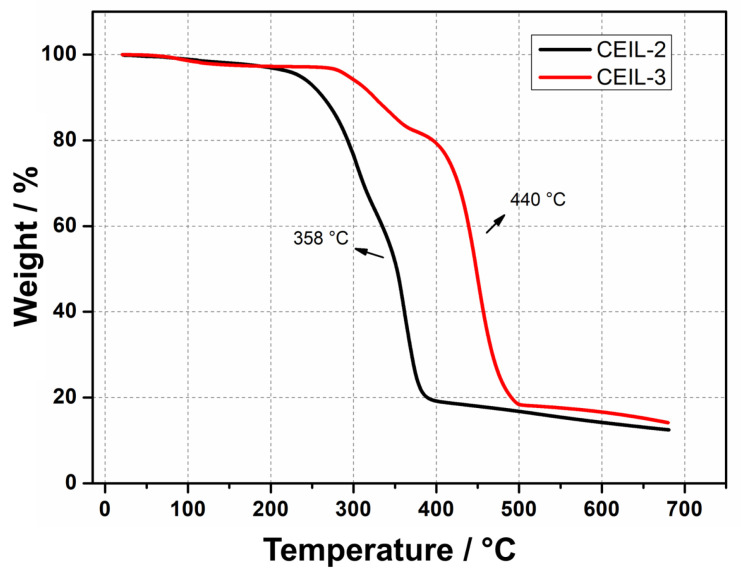
TGA thermograms of CEIL-2 and CEIL-3 monomers under a heating rate of 10 ${}^{{}^{\circ}}$C·min${}^{-1}$.

The thermal stability of both ILs was assessed by thermogravimetric analysis (TGA) (Figure 4). The results revealed outstanding thermal stability for the two monomers, evidenced by minimal weight loss at about 290 ${}^{{}^{\circ}}$C followed by thermal degradation at 358 and 440 ${}^{{}^{\circ}}$C for CEIL-2 and CEIL-3, respectively. The elevated degradation temperatures support our previous works and further confirm the high thermal stabilities of imidazolium-based ILs bearing Tf${}_{2}$N${}^{-}$ as counter-ion. Not surprisingly, CEIL-3 presented greater thermal stability, probably due to the higher physicochemical stability of ether groups compared to esters (see Figure 4) [20,42].

### 3.3. Predicted Thermo–Mechanical Properties of the Polymerised Samples

**Figure 5:**
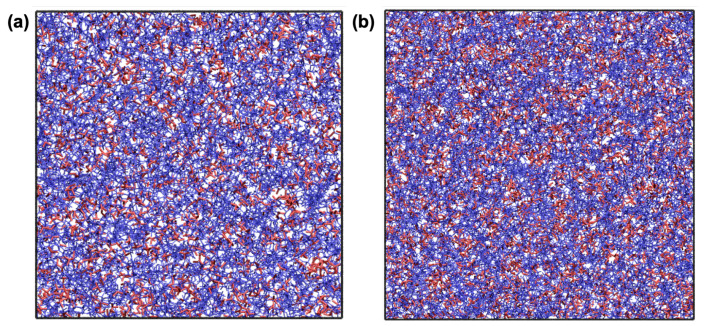
Representative snapshots for (**a**) p-CEIL-2 and (**b**) p-CEIL-3. Colour code: red and blue for Tf${}_{2}$N${}^{-}$ and CEIL-2 (in (**a**)) or CEIL-3 (in (**b**)), respectively. Solid black lines represent simulation cell boundaries.

Upon the completion of the polymerisation process, the resultant samples were cooled from 327 to 27 ${}^{{}^{\circ}}$C to predict the thermal properties. Figure 5 shows representative snapshots for p-CEIL-2 and p-CEIL-3 at 27 ${}^{{}^{\circ}}$C. The snapshots indicate no visible phase separation between the polymer network (i.e., polymerised monomers) and the anions in both systems. In our previous work, we showed that an IL (BmimCl) was phase separated in a typical epoxy system (DGEBA–DETDA) [43]. This phase separation was more visible when the IL content was increased from 10% to 50% by weight. This fact of no phase separation in the CEIL systems can be expected as the polymer network formed between the monomers (cations of the IL) is positively charged. Anions prefer to be found close to the imidazolium rings, the centre of the positive charge of the monomer, preventing any potential phase separation.

**Figure 6:**
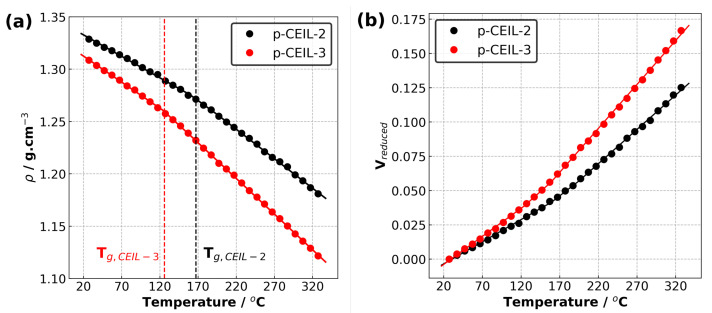
(**a**) Density versus temperature plot and (**b**) reduced volume versus temperature plot for p-CEIL-2 and p-CEIL-3 obtained using isobaric–isothermal molecular dynamics simulations.

Table 2 summarises the properties of the polymerised systems (averaged over three independently generated samples) predicted in our simulations. Figure 6 shows the density of the polymerised samples as a function of temperature for both systems. The structure of CEIL-3 is much more flexible than the structure of CEIL-2 as CEIL-3 has two imidazolium rings connected to each other with long linear chains (Figure 1). This can manifest itself in both the density and T${}_{g}$ of the polymerised sample. It is expected that the density of the p-CEIL-3 results in lower than that of the p-CEIL-2. Our simulation results indicated a density of 1.33 and 1.31 g·cm${}^{-3}$ at 27 ${}^{{}^{\circ}}$C for the p-CEIL-2 and p-CEIL-3, respectively. We also found that the difference in the density of the polymerised CEIL-2 (p-CEIL-2) and the polymerised CEIL-3 (p-CEIL-3) decreased with the decreasing temperature.

Similarly, the T${}_{g}$ value of the p-CEIL-2 appeared to be larger than that of the p-CEIL-3. This can be attributed to the more flexible structure of the CEIL-3 monomer. Experimental studies have shown that the T${}_{g}$ of p-CEIL-2 resulted in between 71 and 117 ${}^{{}^{\circ}}$C depending on the concentration of initiator used and the equipment used for the measurement [20]. Due to the discrepancy between the cooling rate implemented in our simulations and that is accessible in experiments, our predicted T${}_{g}$ values need to be corrected. A common practice is to subtract 3 ${}^{{}^{\circ}}$C for each order of magnitude difference. Therefore, our corrected predicted T${}_{g}$ values were found to be 131 and 90 ${}^{{}^{\circ}}$C for the p-CEIL-2 and p-CEIL-3, respectively. We note here that in our simulations, we generated polymer samples with a degree of conversion of >99%. In experiments, the conversion could be less than 99%. This can explain the slightly larger T${}_{g}$ values obtained in our simulations.

We also predicted the coefficients of volumetric thermal expansions ($\alpha_{v}$) and the coefficients of linear thermal expansions ($\alpha_{l}$) for the glassy region (the region below T${}_{g}$) and amorphous region (the region above T${}_{g}$) for each system [21]. Our results indicate that the $\alpha_{v}$ results in larger in the amorphous region (above T${}_{g}$) than that in the glassy region (below T${}_{g}$) for both systems. This can be attributed to the larger kinetic energy which the systems possess at the temperature higher than T${}_{g}$. The polymer network becomes more flexible, leading to a larger coefficient of thermal expansion. At temperatures lower than T${}_{g}$, the mobility of the polymer network is limited as the kinetic energy of systems is smaller. When we compare the polymerised CEIL systems, our results show that the $\alpha_{v}$ is larger for p-CEIL-3 than for p-CEIL-2 in both the amorphous and glassy regions. This is consistent with the trend in the T${}_{g}$ values and can be ascribed to the more flexible backbone of the CEIL-3 monomer than that of the CEIL-2 monomer.

**Figure 7:**
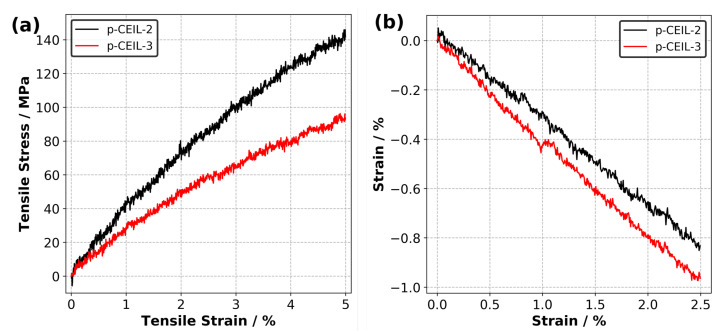
(**a**) Predicted tensile stress versus tensile strain plots and (**b**) strain versus strain plots for p-CEIL-2 and p-CEIL-3 obtained at 27 ${}^{{}^{\circ}}$C.

Figure 7a shows the calculated tensile stress versus tensile strain plots for each polymerised system obtained at 27 ${}^{{}^{\circ}}$C. The Young’s modulus of the samples were obtained by fitting a line to the stress–strain data up to 2.5% of strain. Our results indicate that the Young’s modulus values are 3.7 and 2.5 GPa for the p-CEIL-2 and p-CEIL-3, respectively. Our predicted Young’s modulus values can be larger than those measured experimentally due to the discrepancy between the strain rates used in the simulations and experiments. However, our main interest here is the trend between the Young’s modulus of each system, not the absolute values.

Poisson’s ratio (ν) can be used as a metric to understand the tendency of a material to expand in directions perpendicular to the direction of the strain applied on the material. The larger the Poisson’s ratio, the more the material tends to expand in the lateral directions. Therefore, Poisson’s ratio plays an important role in selecting materials for applications where material requires small/large expansions during deformation. Poisson’s ratio of epoxies varies based on the monomer’s architecture. For example, MD simulations predicted the Poisson’s ratio of a diglycidylether of bisphenol A (DGEBA)–diethyltoluenediamine (DETDA) system to be around 0.38 [24] while the experimental values varied between 0.40 and 0.43 [44]. Smaller Poisson’s ratio values (0.27–0.35) were predicted for epoxy systems based on bio-based monomers, such as divanillyl alcohol-derived epoxy monomers (e.g., diglycidylether of divanillyl alcohol (DiGEDVA)) [45]. Saseendran et al. investigated the dependence of Poisson’s ratio of a commercial epoxy system (Araldite LY5052 epoxy resin cured with Aradur HY5052 hardener) on the degree of curing [46]. These authors showed that the Poisson’s ratio of the epoxy system was ∼0.5 at a low degree of curing (∼50%) and tends to decrease with an increasing degree of curing. When the epoxy system was fully cured (>99%), the Poisson’s ratio achieved its final value of 0.32.

Figure 7b shows the strain versus strain data from which the Poisson’s ratio of the polymerised CEIL systems are calculated. Our results indicate that the p-CEIL-3 possessed a larger ν value (0.40) than the p-CEIL-2 (0.33). This means that when the tensile strain is applied on the p-CEIL-3, it will expand from the lateral sides more when compared with the p-CEIL-2 system. Again, this can be explained by the more flexible backbone of CEIL-3 compared to the CEIL-2. Taken together, the flexibility of the monomer determines the ultimate macroscopic responses of the polymerised sample.

### 3.4. Measured Thermo–Mechanical Properties of the Polymerised Samples

**Figure 8:**
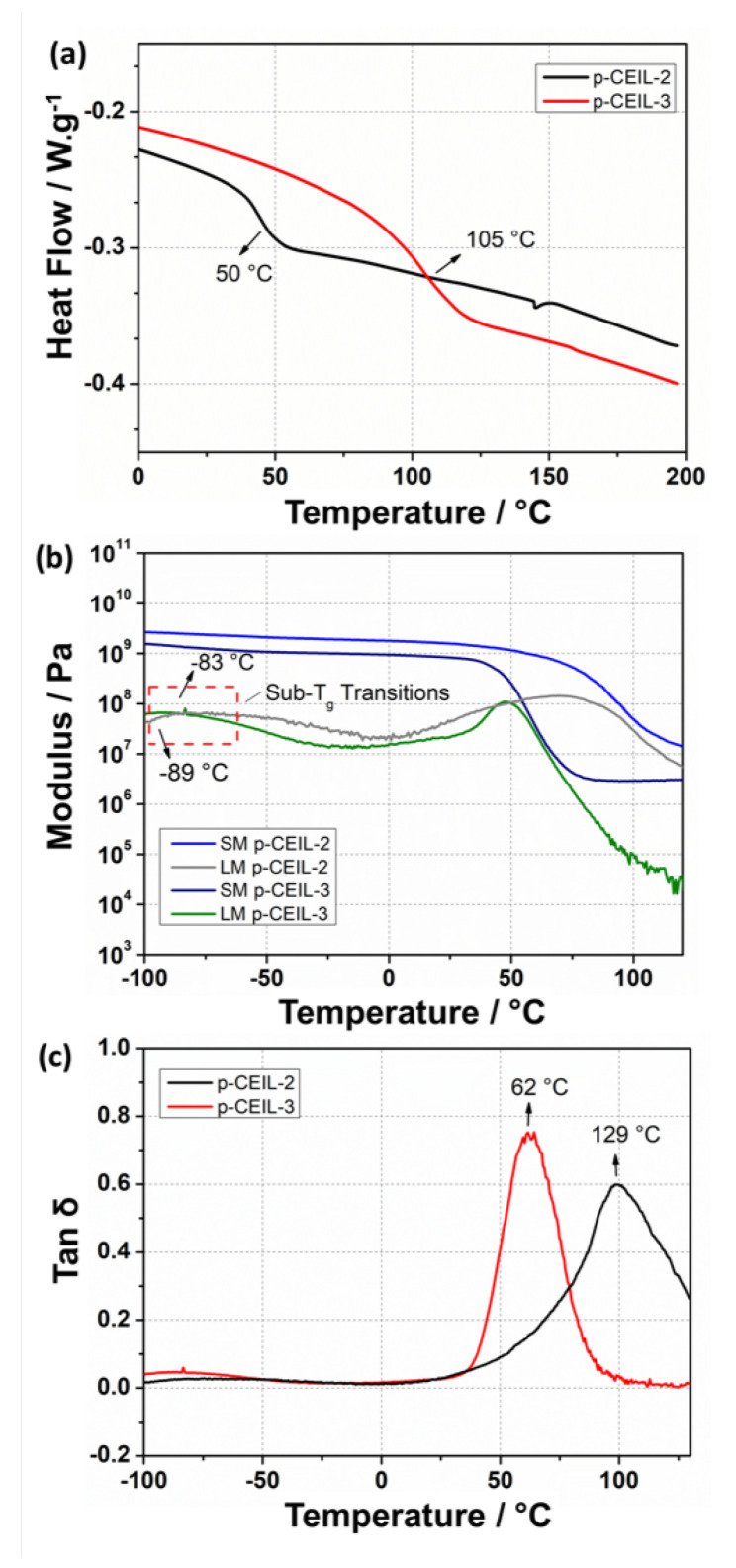
(**a**) DSC thermograms obtained for the epoxy networks based on p-CEIL-2 and p-CEIL-3. (**b**) DMA graph for p-CEIL-2 epoxy network displaying the T${}_{g}$ range from 116 to 140 ${}^{{}^{\circ}}$C. (**c**) DMA graph for p-CEIL-3 epoxy network displaying the T${}_{g}$ range from 50 to 80 ${}^{{}^{\circ}}$C. SM and LM correspond to the storage modulus and loss modulus, respectively.

We also performed experiments to measure the thermo–mechanical properties of the p-CEIL 2 and p-CEIL-3 and reported the results in Figure 8 and Table 3. T${}_{g}$ of the polymerised samples were measured via DSC and DMA. Our results indicate that p-CEIL-2 presented higher T${}_{g}$ values than p-CEIL-3 in both DSC and DMA measurements. Similarly, DMA results show that both G${}^{\prime }$ and G${}^{\prime \prime }$ were larger for the p-CEIL-2 than for the p-CEIL-3 at two different temperatures (25 and 150 ${}^{{}^{\circ}}$C). These results corroborate the theoretical outcomes and agree with the molecular structure of the monomers and their corresponding PIL networks. The CEIL-3 cation possesses an elongated aliphatic chain which ensures free rotation around the C–C bonds, increasing the monomer backbone flexibility.

Additionally, the greater distance between the lateral epoxy groups generates cross-links into the p-CEIL-3 networks more widely spaced, which could also increase the mobility of the polymer chains [20]. This additional mobility for the p-CEIL-3 chains is macroscopically observed by a lower T${}_{g}$ and lower storage modulus values when compared to the p-CEIL-2 network. Interestingly, the resulting DMA graphs display a minor relaxation at −83 and −89 ${}^{{}^{\circ}}$C for p-CEIL-2 and p-CEIL-3 networks, respectively. Such behaviour was already described for different epoxy networks, and it likely originates from the local motion of the moieties such as hydroxyl and diether [47,48].

### 3.5. Mobility of Anions

**Figure 9:**
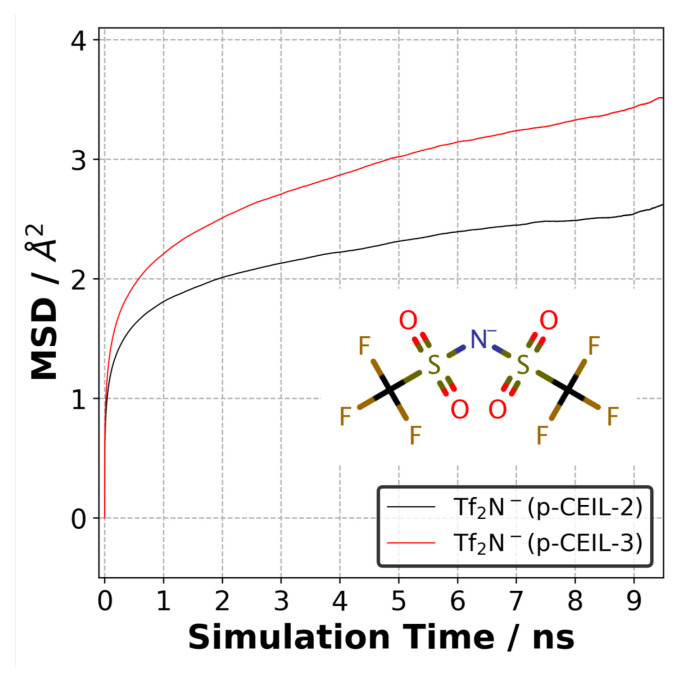
Mean square displacement of anions (Tf${}_{2}$N${}^{-}$) in the p-CEIL-2 and p-CEIL-3 obtained at 27 ${}^{{}^{\circ}}$C.

The positively charged cycloaliphatic cations (or monomers) form the 3D polymer network via covalent bonds. On the other hand, anions, do not contribute to the 3D polymer network, and are able to move freely in the polymer network. To quantify the mobility of anions in both polymer systems, we calculated the mean square displacement (MSD) of anions at 27 ${}^{{}^{\circ}}$C. Figure 9 shows the MSD curves for anions averaged over three independently generated samples for each system. Although the equilibrium density of the polymerised systems is similar, the difference in the mobility of anions is substantial. Our results indicate that the anions are more mobile in the p-CEIL-3 than in the p-CEIL-2. This can be attributed to the more flexible structure of the CEIL-3 monomer.

Our results also show that the mobility of anions in the polymerised samples was similar in all the three directions (see SI). This means that the polymer network formed between the cationic monomers evolved isotropically. It did not lead to the formation of direction-biased polymer network. We did not calculate the diffusion coefficient of the anion in the polymer as the MSD curves did not reach a diffusive regime. The R${}^{2}$ value of the log–log plot of MSD plots gave us a value of ∼0.20. This means that longer simulations need to be performed to observe the anions’ mobility in the diffusive regime.

## 4. Conclusions

In this work, we modelled the polymerisation of CEILs and predicted their thermo–mechanical properties. The liquid mixtures of cations (monomers)–anions were adequately equilibrated, and the molecular-level structuring in the liquid mixtures was studied via RDFs. Our results indicated that the minimum distance between the reactive atomic sites of the cation and nitrogen atom of the anion was ∼4.9 Å. The thermal stability of ILs was assessed via thermogravimetric analysis (TGA), and our results indicated a thermal degradation at 358 and 440 ${}^{{}^{\circ}}$C for CEIL-2 and CEIL-3, respectively, indicating high thermal stabilities of CEILs. Our in situ polymerisation protocol was then applied to generate polymerised samples. We visually inspected our polymerised samples and observed no visible phase separation between the polymer network and freely moving anions. The densities of the p-CEIL-2 and p-CEIL-3 were predicted very similar. This can be attributed to the similar structures of the backbone of the monomers. We found in our simulations and experiments that the p-CEIL-2 possessed a higher T${}_{g}$ than p-CEIL-3 (∼30%). This can be due to the flexible backbone of the CEIL-3 monomer. Similarly, p-CEIL-2 exhibited a Young’s modulus ∼30% larger than that of p-CEIL-3. The experimental results showed that the G${}^{\prime }$ and G${}^{\prime \prime }$ for p-CEIL-2 were 3.3 and 14.7 times larger than those for p-CEIL-3. The mobility of anions in the p-CEIL-2 and p-CEIL-3 is slow, which can be exploited for applications requiring the long-term mobility of anions. The computational procedure reported herein will enable us to advance our knowledge on the novel poly(ionic liquids) (PILs) based on epoxidised cycloaliphatic groups with a molecular-level resolution. In addition, our computational procedure can be exploited to screen numerous PILs, and the outcome of simulations results could be used as input to a machine learning pipeline to explore new materials with optimised ultimate performance.

## Figures and Tables

**Table 1:** Number of monomers and anions as well as the total number of atoms in each liquid system are reported. The equilibrium density and equilibrium cubic cell size were obtained at 227 ${}^{{}^{\circ}}$C and 1 atm.

	CEIL-2	CEIL-3
No. of monomers	400	400
No. of anions	400	800
No. of atoms	30,800	51,200
Cubic cell size/Å	74.6	89.1
Liquid density (227 ${}^{{}^{\circ}}$C)/g·cm${}^{-3}$	1.102	1.119

**Table 2:** Predicted properties of the polymerised samples (averaged over three independently generated samples).

	p-CEIL-2	p-CEIL-3
Density (27 ${}^{{}^{\circ}}$C)/g·cm${}^{-3}$	1.330	1.310
T${}_{g}$/${}^{{}^{\circ}}$C	131	90
CVTE × $10^{4}$/${}^{{}^{\circ}}$C${}^{-1}$ (below T${}_{g}$)	3.22	4.09
CVTE × $10^{4}$/${}^{{}^{\circ}}$C${}^{-1}$ (above T${}_{g}$)	5.01	6.41
Young’s modulus/GPa	3.7	2.5
Poisson’s ratio/-	0.33	0.40

**Table 3:** Experimentally measured thermo–mechanical properties of the polymerised samples.

	p-CEIL-2	p-CEIL-3
T${}_{g},DSC$/${}^{{}^{\circ}}$C	105	50
T${}_{g},DMA$/${}^{{}^{\circ}}$C	129	62
G${}^{\prime }$${}_{25^{{}^{\circ}}C}$/GPa	0.827	0.250
G${}^{\prime }$${}_{150^{{}^{\circ}}C}$/GPa	0.014	0.003
G${}^{\prime \prime }$${}_{25^{{}^{\circ}}C}$/GPa	1.507	0.102
G${}^{\prime \prime }$${}_{150^{{}^{\circ}}C}$/MPa	5.162	0.007
tanδ${}_{25^{{}^{\circ}}C}$/-	0.027	0.409
tanδ${}_{150^{{}^{\circ}}C}$/-	0.380	0.002

## Abbreviations

The following abbreviations are used in this manuscript:

IL	Ionic liquid
MD	Molecular dynamics
SA	Simulated annealing
MSD	Mean square displacement
SSC	Stress–strain curve
